# Function of ABA in Stomatal Defense against Biotic and Drought Stresses

**DOI:** 10.3390/ijms160715251

**Published:** 2015-07-06

**Authors:** Chae Woo Lim, Woonhee Baek, Jangho Jung, Jung-Hyun Kim, Sung Chul Lee

**Affiliations:** 1 Department of Life Science (BK21 program), Chung-Ang University, Seoul 156-756, Korea; E-Mails: gelslim@hanmail.net (C.W.L.); caly1006@gmail.com (W.B.); jangho12345@naver.com (J.J.); 2 Department of Home Economics Education, Chung-Ang University, Seoul 156-756, Korea

**Keywords:** abscisic acid (ABA), ABA receptor, biotic and abiotic stresses, PP2C, SnRK2, stomatal immunity

## Abstract

The plant hormone abscisic acid (ABA) regulates many key processes involved in plant development and adaptation to biotic and abiotic stresses. Under stress conditions, plants synthesize ABA in various organs and initiate defense mechanisms, such as the regulation of stomatal aperture and expression of defense-related genes conferring resistance to environmental stresses. The regulation of stomatal opening and closure is important to pathogen defense and control of transpirational water loss. Recent studies using a combination of approaches, including genetics, physiology, and molecular biology, have contributed considerably to our understanding of ABA signal transduction. A number of proteins associated with ABA signaling and responses—especially ABA receptors—have been identified. ABA signal transduction initiates signal perception by ABA receptors and transfer via downstream proteins, including protein kinases and phosphatases. In the present review, we focus on the function of ABA in stomatal defense against biotic and abiotic stresses, through analysis of each ABA signal component and the relationships of these components in the complex network of interactions. In particular, two ABA signal pathway models in response to biotic and abiotic stress were proposed, from stress signaling to stomatal closure, involving the pyrabactin resistance (PYR)/PYR-like (PYL) or regulatory component of ABA receptor (RCAR) family proteins, 2C-type protein phosphatases, and SnRK2-type protein kinases.

## 1. Function of Abscisic Acid (ABA) in Response to Biotic and Abiotic Stresses

Plants are sessile organisms and therefore they constantly encounter diverse biotic and abiotic stresses, including various pathogens, drought, and high salinity. These stresses affect plant growth and development and can severely impair crop production. The plant hormone abscisic acid (ABA) functions as a chemical signal in response to environmental stresses. Stress signals are converted to ABA and this triggers the activation of a number of plant physiological and developmental processes, thereby inducing adaptation to the stress conditions [1,2,3]. Defense responses to biotic and abiotic stress have been extensively investigated [4,5,6,7]. In the present review, we focus on recent research into ABA responses to stomatal defense, and crosstalk of biotic and abiotic responses through the regulation of stomatal movement.

### 1.1. Function of ABA in the Regulation of Stomatal Movement

Biotic and abiotic stresses adversely affect plant growth and induce severe losses in agricultural crop production. Plants lose water primarily by gaseous exchange through the stomata on their leaves. ABA is a key hormone that regulates water status and stomatal movement. Under drought conditions, plants produce and accumulate increased amounts of ABA in the guard cells, and this induces stomatal closure to conserve water. The cellular and molecular mechanisms underlying ABA-induced stomatal closure have been extensively investigated [4,5,8,9,10,11,12,13]. ABA biosynthesis and catabolism are known to be major determinants of endogenous ABA levels in plant cells [14,15]. The 9-*cis*-epoxycarotenoid dioxygenase (*NCED*) genes and cytochrome P450 *CYP707A* genes encode key enzymes for ABA biosynthesis and ABA catabolism, respectively. The *NCED3* gene is induced by drought stress and it upregulates endogenous ABA levels in overexpressed transgenic plants, thereby leading to lower transpiration rates [16,17,18,19,20]. Regarding ABA catabolism, the expression of *CYP707A1* to *CYP707A4* genes trigger ABA 8′-hydroxylation [21,22]. Among these four members of the *CYP707A* gene family, the transcripts of *CYP707A3* were shown to be the most highly accumulated. Moreover, the results of genetics analysis revealed that the *cyp707a3-1 Arabidopsis* mutant accumulated higher endogenous ABA levels and exhibited a reduced transpiration rate, thereby resulting in a phenotype exhibiting enhanced tolerance to drought stress [21].

Stomatal control is regulated by a number of environmental factors, including CO_2_ level, light, and biotic and abiotic stresses. Guard cell turgor pressure is a key parameter regulating stomatal opening and closure, and it in turn is mediated by ionic fluxes via cation and anion channels anchored in the guard cell membrane. Under conditions of biotic and abiotic stresses, ABA functions as a chemical messenger that induces stomatal closure through the activation and inactivation of ion channels by protein kinases and phosphatases [23,24,25,26,27,28,29,30,31,32,33,34,35,36]. The entire sequence of ABA signal transduction, from ABA receptors to stomatal closure, will be discussed later in this review.

### 1.2. Role of Stomatal Immunity via Restriction of Pathogen Entry

**Figure 1:**
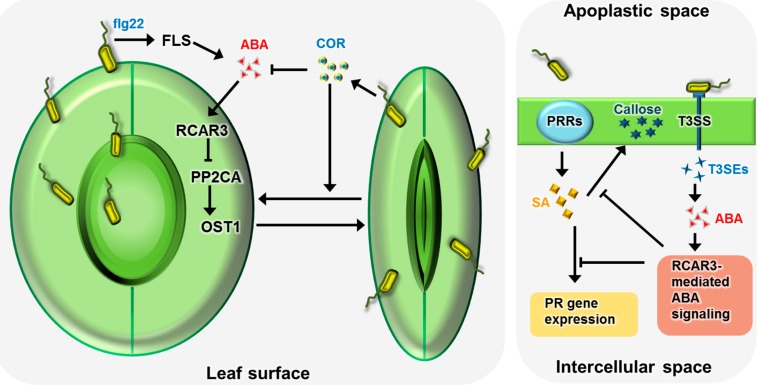
Schematic representation of a possible mechanism underlying the antagonistic and synergistic role of abscisic acid (ABA) signaling in plant defense response at the pre-invasive and post-invasive stages of *Arabidopsis thaliana*–*Pseudomonas syringae* interaction. At the pre-invasive stage, ABA signaling plays a synergistic role in plant resistance to *P. syringae* attack. Stomata constitute a major route of bacterial entry. Pathogen-associated molecular pattern (PAMP)-induced ABA signaling in the guard cells promotes stomatal closure and actively blocks *P. syringae* invasion [44,48]. This stomatal immunity involves the ABA receptor–2C-type protein phosphatase–open stomata 1 (RCAR-PP2C-OST1) complex acting as a core component of ABA signaling [12,44]. The phytotoxin coronatine (COR) is a virulence factor produced by *P. syringae* and it can compromise PAMP-induced stomatal defense by suppressing PAMP-induced ABA signaling and promoting stomatal reopening [12,44,49]. In contrast, ABA signaling plays an antagonistic role in post-invasive defense response. *Pseudomonas syringae* type III secreted effector (T3SE) proteins upregulate ABA biosynthesis and also the signaling pathways, thereby inhibiting the plant defense response [44,50]. In this process, ABA signaling antagonizes salicylic acid (SA)-mediated pathogenesis-related (PR) gene expression and callose deposition [12,48,50].

Recently, several studies have demonstrated that ABA plays a crucial role in pathogen response and that ABA signaling overlaps considerably between biotic stress resistance and abiotic stress tolerance. Plants possess physical and biochemical defense barriers that effectively protect them from diverse pathogens. Various foliar pathogens such as bacteria, fungi, and viruses are known to disrupt stomatal movement in order successfully to infect plants [37,38,39]. The first line of defense is the recognition of the evolutionary conserved pathogen materials—termed the pathogen-associated molecular pattern (PAMP)—by plant pattern recognition receptors (PRRS), thereby leading to PAMP-triggered immunity (PTI) [40,41,42]. The second line of defense is the recognition of effectors through plant resistance (R) proteins, thereby leading to effector-triggered immunity (ETI) [40,43]. Melotto *et al.* [44] showed that stomata constitute not only a path for transpiration, but also a port for pathogen entry. Thus, stomata play a crucial role in the plant immune response, and the regulation of stomatal movement is an early stage of defense mechanism against pathogen infection (Figure 1). Conversely, most pathogens have evolved mechanisms that allow them to overcome or circumvent plant physical barriers, including stomatal closure, thereby enabling them to successfully infect plants [40].

### 1.3. Role of Stomatal Closure in Drought Responses

The primary function of stomatal closure is to prevent water loss, thereby inducing drought tolerance under conditions of osmotic stress. The regulation of stomatal closure is important not only for the defense mechanism to prevent invasion of bacterial pathogens, but also for water conservation. Drought is a major osmotic stress that affects plant growth and development, thereby leading to severe losses in agricultural crop production. Stomatal opening and closure affect various physiological processes and properties, such as photosynthesis and water status. Plants gain water through their roots and lose water primarily via the stomata on their leaves. If the amount of water lost through the leaves exceeds the amount taken up by the roots, plant tissues can be damaged, thereby resulting in cell death. Under drought conditions, ABA is produced or accumulated in the guard cells that surround the stomata, and this induces stomatal closure, thereby conserving water [11,45]. The cellular and molecular regulation of stomatal opening and closure under drought conditions has been extensively investigated and reviewed [4,5,8,9,10,13]. ABA-controlled processes are necessary for plant survival, and ABA-deficient mutants are susceptible to water stress [3,46,47].

Guard cell turgor pressure is a key parameter regulating stomatal control, and this in turn is mediated by ionic fluxes across the cell membranes through K^+^ and anion channels. Consequently, these ion channels constitute the major target for regulation by a number of environmental factors, including light, dark, drought, CO_2_ levels, *etc.* Under drought conditions, ABA serves as a chemical messenger that induces stomatal closure through second messengers, such as ROS, nitric oxide, Ca^2+^, and protein kinases; these messengers further target the ion channels [23,24,25,26,27,28,29,30,31,32,33,34,35,36]. The entire sequence of ABA signal transduction, from ABA receptors to stomatal closure, will be discussed later in this review.

## 2. ABA Signal Transduction Pathway

### 2.1. Perception and Transfer of the ABA Signal

Previous studies have identified positive and negative regulators of ABA signaling, from ABA receptors to ion channels [8,11,24,26,51,52,53,54,55,56,57,58]. To initiate ABA signal transduction, ABA receptors must be present and must deliver the ABA signal to the downstream pathway in plant cells. Several types of ABA receptor have been identified [9,51,57,59]. ABA-binding activities are known to be present in several locations, including the cell membrane and cytoplasm, indicating that more than one ABA receptor may exist within a single cell [60,61,62]. However, with the exception of the pyrabactin resistance (PYR)/PYR-like (PYL) or regulatory component of ABA receptor (RCAR) family proteins, few studies of ABA receptor function have been conducted. Therefore, here we will focus on the function of PYR/PYL/RCAR proteins in relation to other positive and negative regulators of ABA signaling, including 2C-type protein phosphatases (PP2Cs) and SnRK2 (SNF1-related kinase 2)-type protein kinases.

The PYR/PYL/RCAR protein family comprises 14 members, all of which function in ABA perception and signaling [9,51,57,63,64]. The results of genetic analysis using triple (*pyr1*:*pyl1*:*pyl4*), quadruple (*pyr1*:*pyl1*:*pyl2*:*pyl4*), and sextuple (*pyr1*:*pyl1*:*pyl2*:*pyl4*:*pyl5*:*pyl8*) mutants revealed that these mutants displayed ABA-insensitive phenotypes during the germinative, seedling, and adult stages [51,64]. In contrast, the single mutant did not display any altered phenotype to ABA [57,65]. Moreover, the results of structural analyses showed that the PYR/PYL/RCAR proteins harbor a ligand-binding pocket, which may function as an ABA- and group A PP2C-binding site [66,67,68,69,70]. Several previous studies have demonstrated that group A PP2Cs function as negative regulators of ABA signaling [13,71,72,73]; moreover, PYR/PYL/RCAR proteins directly inhibit phosphatase activity of PP2Cs *in vitro* [51,74]. Thus, these PP2Cs constitute direct targets of PYR/PYL/RCAR proteins in the ABA signaling pathway. The results of *in vitro* and *in vivo* interaction assays between PYR/PYL/RCAR proteins and their target PP2Cs showed that ABA enhances these interactions; in contrast, other interactions are independent of ABA [12,51,58,63]. These findings imply that ABA binding induces structural changes in PYR/PYL/RCAR proteins and that the ABA-bound PYR/PYL/RCAR proteins can interact tightly with PP2Cs, or that ABA does not change the protein structures and that PP2Cs bind only to the PYR/PYL/RCAR-ABA complex. Whichever is the case, the interactions between PYR/PYL/RCAR proteins and their target PP2Cs induce the activation of downstream targets of the group A PP2Cs, including SnRK2 protein kinases (SnRK2.2, SnRK2.3, SnRK2.6) and the S-type anion channel (SLAC1); these in turn play key roles in the regulation of transcriptional response and stomatal closure [26,56,58]. Several studies have shown that these ABA receptors have a functional redundancy in ABA signaling. In addition, with the exception of PYL13, all the PYR/PYL/RCAR protein family members are able to induce ABA-responsive genes [52]. However, the expression patterns differ from each other, indicating that the functions of PYR/PYL/RCAR proteins and their *in vivo* downstream target specificity may vary markedly.

The ubiquitination system via the 26S proteasome pathway plays a crucial role in hormone signaling, including the perception of auxin [75,76,77], jasmonate [78], gibberellins [79], and ABA [80,81,82,83,84]. Ubiquitination is a key post-translational modification performed by the sequential action of three enzymes—ubiquitin activating enzyme (E1), ubiquitin-conjugating enzyme (E2), and ubiquitin ligase (E3). In this process, E3 ligase determines and recruits substrate proteins. In the ABA signaling pathway, several E3 ligases induce changes in plant responses to ABA, by degradation of positive and negative regulators of ABA signaling [85]. Many E3 ligases related to ABA signaling have been identified in *Arabidopsis* and rice [81,83,84,86,87,88,89,90,91,92]; however, only a few substrate proteins have been identified using interaction assays. In addition, most target proteins are restricted to the final stage of ABA signal transduction; these include transcription factors, which regulate the expression of ABA-responsive genes, for example, ABF2 (ABRE-binding bZIP transcription factor 2) (ubiquitinated by ARIA (arm repeat protein interacting with ABF2)), ABI3 (abscisic acid-insensitive 3) (ubiquitinated by AIP2 (ABI3-interacting protein 2)), ABI5 (ABA insensitive 5) (ubiquitinated by KEG (KEEP ON GOING), DWA1 (DWD hypersensitive to ABA1), DWA2, and ABD1 (ABA-hypersensitive DCAF1)), and ERF53 (ethylene response factor 53) (ubiquitinated by RGLG1 (RING domain ligase 1) and RGLG2) [83,91,92,93,94,95]. However, recent studies have demonstrated that ABA receptors (PYR1, PYL4, PYL8, and PYL9) are ubiquitinated by CRL4 (Cullin4-RING E3 ligase) and RSL1, thereby leading to proteasomal degradation and subsequent attenuation of ABA signaling [82,84].

In response to biotic and abiotic stresses, reactive oxygen species (ROS), such as superoxide radical, hydrogen peroxide (H_2_O_2_), and nitric oxide (NO) are produced and involved in defense mechanisms [40,96,97]. The ROS function as second messengers of ABA. NADPH (nicotinamide adenine dinucleotide phosphate) oxidases, which referred to as respiratory burst oxidases (RBOH), are induced by biotic and abiotic stresses [96,98,99]. The AtrbohD and AtrbohF are associated with production of ROS in response to ABA [100] and pathogen infection, such as *Pseudomonas syringae* and *Hyaloperonospora Arabidopsis* [98]. The *atrbohD*/*atbohF* double mutant exhibits impaired ABA signaling phenotypes including stomatal closure, ROS production and cytosolic Ca^2+^ induction [100]. The *atrbohD*, *atbohF* and *atrbohD*/*atbohF* mutants also show reduced ROS production and reduced cell deateh in response to pathogen infection. Moreover, these mutants are more susceptible to bacterial pathogen *P*. *syringae* pv. *tomato* DC3000 (avrRpm1) and the oomycete parasite *H. Arabidopsis* [98].

### 2.2. Phosphorylation and Dephosphorylation Events in ABA Signaling Transduction

Several protein kinases and phosphatases are involved in protein phosphorylation and dephosphorylation events in ABA signaling in plants [26,56,58,101,102]. The functions of the SnRK2-type protein kinases in relation to ABA were first elucidated using PKABA1 (ABA-induced Ser/Thr protein kinase 1) in wheat [103]. The kinase activity of PKABA1 is induced by ABA and is involved in the phosphorylation and activation of the transcription factor TaABF1 (*Triticum aestivum* ABRE-binding bZIP transcription factor 1), which induces ABA-responsive gene expression in wheat and barley [103,104,105,106]. Subsequent genetic studies identified open stomata 1 (OST1)/SnRK2.6, which is a Ser/Thr protein kinase, in *Arabidopsis* [107]. ABA did not induce OST1 expression but did promote kinase activity of this protein [107,108]. The *ost1* mutant exhibits an ABA-insensitive and drought-sensitive phenotype, which is characterized by a high level of leaf water loss due to permanently open stomata and a minimal ABA-responsive gene expression [107,108]. OST1 is able to interact with and phosphorylate ABF2 and ABF3, which are transcription factors that bind to ABA-responsive elements (ABRE) and control ABA-responsive gene expression [7,52,109,110]. Furthermore, phosphorylation of ABF3 via OST1 creates the 14-3-3 protein binding motif and this stabilizes ABF3. ABFs, including ABF3 and ABF4, are also involved in drought stress tolerance through the regulation of stomatal closure, thereby leading to a decreased transpirational rate in *Arabidopsis* [66]. Two other SnRK2-type kinases—SnRK2.2 and SnRK2.3—are induced by ABA, and the *snrk2.2*/*snrk2.3* double mutant exhibits an ABA-insensitive phenotype [53,111]. A triple mutant (*snrk2.2*/*snrk2.3*/*ost1*) displays strongly ABA-insensitive and drought-sensitive phenotypes, implying that these kinases have functional redundancy in ABA and drought signaling [54,109,112]. Moreover, OST1 functions as a positive regulator of freezing tolerance by regulating CBF transcription factors via phosphorylation of ICE1 (inducer of CBF expression 1) [102].

In *Arabidopsis*, 76 out of 112 phosphatase genes have been identified as PP2Cs [71,113]. These include nine group A PP2Cs, of which six are involved in the regulation of ABA signaling [71,114]. The results of genetic analysis revealed that group A PP2Cs—ABI1 (ABA-insensitive 1), ABI2, HAB1 (hypersensitive to ABA 1), HAB2, AHG1 (ABA-hypersensitive germination 1), and PP2CA (protein phosphatase 2CA)—are negative regulators of ABA signaling and that loss-of-function mutants in *Arabidopsis* exhibit an ABA-hypersensitive phenotype during seed germination [13,72,73,115,116,117,118,119,120,121]. The PP2C function related to ABA was first demonstrated using the dominant mutants *abi1-1* and *abi2-1*, which display an ABA-insensitive phenotype during seed germination, seedling growth, and stomatal closure [122,123,124,125].

**Figure 2:**
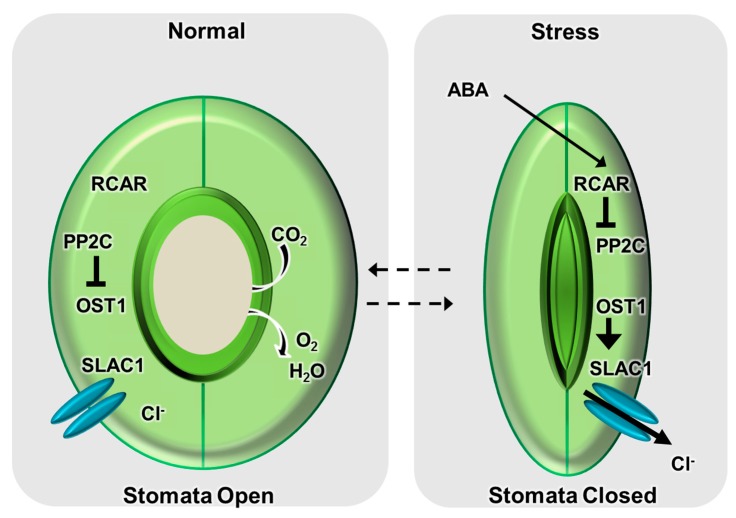
Simplified overview of stomatal movement via the abscisic acid (ABA) signaling pathway. Under normal conditions, 2C-type protein phosphatase (PP2C) family members, which are negative regulators of ABA signaling, suppress open stomata 1 (OST1) kinase activity via physical interaction, leaving the S-type anion channel (SLAC1) with basal activity. Under conditions of stress, including drought, the ABA concentration in leaves increases rapidly [9,10]. ABA perception occurs via regulatory component of ABA receptor (RCAR) family members functioning as ABA receptors in combination with PP2Cs [9,135]. The formation of the RCAR–PP2C complex breaks the PP2C–OST1 complex, thereby releasing active OST1 kinase from inhibition. In turn, OST1-mediated phosphorylation induces activation of the SLAC1 channel, thereby releasing anions and depolarizing the membrane [26]. This depolarization induces a further drop in turgor and closure of the stomatal pores.

Several studies have evaluated the functional and physical interactions between PYR/PYL/RCAR, PP2C, and SnRK2 [26,51,56,57,58,126,127]. These interactions were initially suggested to occur via physical interactions between OST1 and ABI1/ABI2 [128]. More recently, studies have identified several PYR/PYL/RCAR-PP2C-SnRK2 interactions that clearly function in ABA and stress signaling [12,64,102]. For example, the physical interaction between RCAR2 and PP2CA induces inactivation of OST1 kinase, thereby leading to downregulation of SLAC1 activity in guard cells [58]. These PP2Cs have a functional redundancy in ABA signaling; hence, double or triple mutants have strongly ABA-insensitive phenotypes [53,115,126]. The triple mutant *abi1-2/hab1-1/pp2ca-1* displays constitutive activation of SnRK2-type kinases [52], suggesting that the ABA-dependent activation of SnRK2-type kinases is derived from removal of the inhibitory effect of PP2Cs. The ABA-dependent physical interactions of PYR/PYL/RCAR induce the activation of SnRKs, thereby promoting transfer of the ABA signal to the downstream pathway (Figure 2) [51,52,57,127].

### 2.3. ABA Signal Transduction in Biotic Stress

The regulation of stomatal closure constitutes an important layer of the PAMP-triggered defense mechanism at the pre-invasive level. ABA-mediated stomatal closure inhibits pathogen entry to the apoplastic space; hence, ABA functions as a positive regulator of the defense response (Figure 1). Plant recognition of PAMPs via PRRs leads to stomatal closure, thereby restricting pathogen entry [11,44,129,130,131,132]. Several studies have suggested that ABA is associated with PAMP-triggered stomatal closure. Melotto *et al.* [44] used the *ost1* (*snrk2.6*) mutant, which is a positive regulator of ABA [107], and the ABA-deficient *aba3-1* mutant [133] to show that stomatal closure is not induced by PAMPs. Conversely, overexpression of ABA receptors, such as *RCAR3*, *RCAR4*, and *RCAR5*, which function as positive regulators of ABA, induced the maintenance of stomatal closure during *Pseudomonas syringae* pv. *tomato* (*Pst*) DC3000 inoculation and PAMP treatment, thereby leading to enhanced resistance to *Pst* DC3000 [12]. Moreover, *pp2ca* and *hab1*, the group A 2C type protein phosphatases, mutants were positively correlated with stomatal immunity, similar to overexpression of *RCAR3* and *RCAR4*, respectively [12,134].

After pathogen entry to the apoplastic space, ABA affects pathogen response by interacting with defense-related hormones such as salicylic acid (SA), jasmonic acid (JA), and ethylene, and by acting antagonistically towards these hormones [38,44,65,136,137]. Typically, SA is associated withresistance to biotrophic and hemibiotrophic pathogens and expression of acidic pathogenesis-related (PR) genes; whereas JA and ethylene are associated with resistance to necrotrophic pathogens and expression of basic PR genes [138,139,140]. Previous studies have suggested that ABA signaling transduction negatively regulates SA-mediated pathogen responses. For instance, overexpression of *RCAR3*, which functions as a positive regulator of ABA, enhanced susceptibility to virulent bacterial pathogens, by suppressing SA-mediated defense responses such as callose deposition and gene expression at the post-invasive level (Figure 1) [12,135]. In addition, overexpression and mutation of the *NCED* and *ABA3* genes, which are involved in ABA biosynthesis, conferred susceptible and resistant phenotypes, respectively, to *Pseudomonas syringae* pv. *tomato* (*Pst*) DC3000 [67]. Previous genetic studies have investigated the negative effect of ABA on JA- and ethylene-dependent pathogen resistance. The *OsMPK5* (*Oryza sativa* mitogen-activated protein kinase 5) is induced by ABA and by biotic and abiotic stresses [141]. Overexpression of *OsMPK5* enhances the ABA level, but reduces ethylene accumulation, thereby resulting in a phenotype exhibiting enhanced susceptibility to the bacterial and fungal pathogens *Burkholderia glumae* and *Magnaporthe oryzae*, respectively [137,141]. However, it has been shown that ethylene functions as both positive and negative regulators of stomatal opening and closing, depending on the tissue and environmental conditions [96,142,143,144,145]. Thus, cooperated regulation of ABA and ethylene signaling in response to the prevailing environmental conditions is necessary for fine-tuning of stomatal opening and closure, thereby optimizing gaseous exchange for photosynthesis while limiting transpirational water loss and/or pathogen invasion [146,147]. Based on the above findings, we propose that PAMP-induced stomatal closure requires the activation of the ABA signal transduction pathway in guard cells and that ABA functions as a positive regulator of disease resistance at the pre-invasive level. In contrast, defense hormone-triggered resistance inhibited by ABA in the apoplastic space and ABA has a negative effect on disease resistance at the post-invasive level.

## 3. Conclusions and Perspective

The plant hormone ABA is involved in several biotic and abiotic responses and is associated with the regulation of complex signal transductions including seed dormancy, growth and development. Although a diversity of studies have been performed on ABA and its role in defense responses to biotic and abiotic stresses, no clear model of ABA on these stresses has been proposed, nor have its relation between biotic and abiotic stress been adequately determined. Several key components compose the ABA signaling pathway and each component plays a role as a positive and negative regulator in each step. Several members of the group A PP2Cs and SnRK2s ABA receptors are known to exist. Thus, many possible interactions can occur and each interaction has the potential to regulate downstream targets. Consequently, regulatory fine-tuning is extremely complex. Further detailed studies are required to clarify the complex network of interactions occurring in biotic and abiotic signaling pathways. The results of these studies will facilitate the development of genetically engineering agricultural crops with strong resistance to environmental stresses.

## Abbreviations

ABA: Abscisic acid
ABRE: ABA-responsive elements
CRL4: Cullin4-RING E3 ligase
E1: Ubiquitin-activating enzyme
E2: Ubiquitin-conjugating enzyme
E3: Ubiquitin ligase
ETI: Effector-triggered immunity
JA: Jasmonic acid
NCED: 9-*cis*-epoxycarotenoid dioxygenase
NO: Nitric oxide
OST1: Open Stomata 1
PAMP: Pathogen-associated molecular pattern
PP2C: 2C-type protein phosphatases
PR: Pathogenesis related
PRR: Pattern recognition receptor
PTI: PAMP triggered immunity
PYR: Pyrabactin resistance
RCAR: Regulatory component of ABA receptor
ROS: Reactive oxygen species
SA: Salicylic acid
SLAC: *S*-type anion channel
SnRK: SNF related kinase
